# Supplementary material for: Differentiating between Enterococcus faecium and Enterococcus lactis by Matrix-Assisted Laser Desorption Ionization Time-of-Flight Mass Spectrometry
Source: Foods. 2022 Apr 5;11(7):1046. doi: 10.3390/foods11071046 (PMC8997568; doi:10.3390/foods11071046)
Supplement: Supplementary file 1 [file foods-11-01046-s001.zip › foods-1662740-supplementary.pdf]

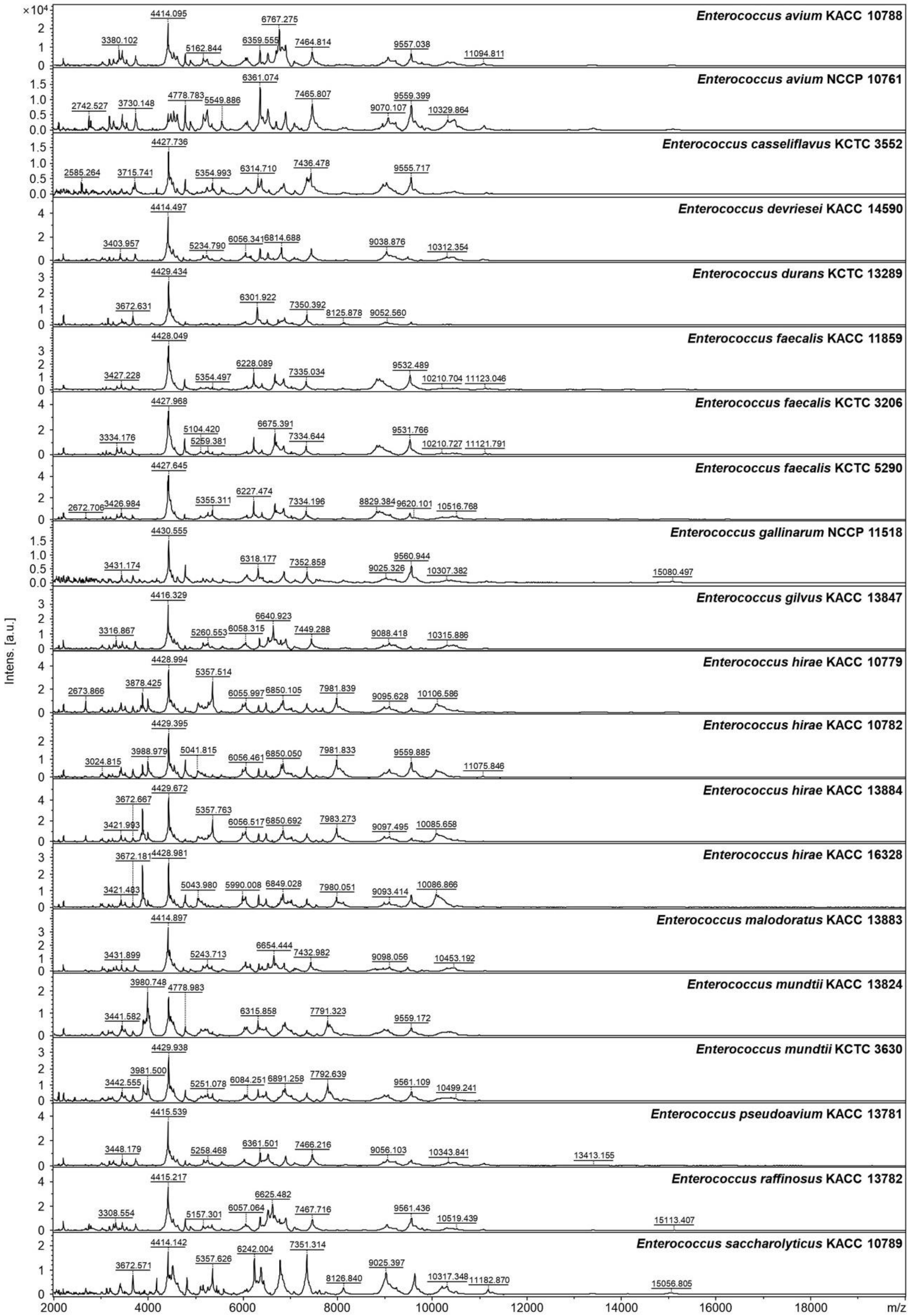

Figure S1. The mass spectra of reference strains of *E. avium*, *E. casseliflavus*, *E. devriesei*, *E. durans*, *E. faecalis*, *E. gallinarum*, *E. gilvus*, *E. hirae*, *E. malodoratus*, *E. mundtii*, *E. pseudoavium*, *E. raffinosus*, *E. saccharolyticus*; m/z, mass-to-charge ratio; a.u., arbitrary units.
